# Nucleic acid vaccines: innovations, efficacy, and applications in at-risk populations

**DOI:** 10.3389/fimmu.2025.1584876

**Published:** 2025-05-14

**Authors:** Emily N. Konopka, Arden O. Edgerton, Michele A. Kutzler

**Affiliations:** ^1^ Drexel University College of Medicine, Department of Microbiology and Immunology, Philadelphia, PA, United States; ^2^ Drexel University College of Medicine, Department of Medicine, Division of Infectious Diseases and HIV Medicine, Philadelphia, PA, United States

**Keywords:** nucleic acid vaccines, mRNA vaccine, DNA vaccine, aging, immunosenescence

## Abstract

For more than two centuries, the field of vaccine development has progressed through the adaptation of novel platforms in parallel with technological developments. Building off the advantages and shortcomings of first and second-generation vaccine platforms, the advent of third-generation nucleic acid vaccines has enabled new approaches to tackle emerging infectious diseases, cancers, and pathogens where vaccines remain unavailable. Unlike traditional vaccine platforms, nucleic acid vaccines offer several new advantages, including their lower cost and rapid production, which was widely demonstrated during the COVID-19 pandemic. Beyond production, DNA and mRNA vaccines can elicit unique and targeted responses through specialized design and delivery approaches. Considering the growth of nucleic acid vaccine research over the past two decades, the evaluation of their efficacy in at-risk populations is paramount for refining and improving vaccine design. Importantly, the aging population represents a significant portion of individuals highly susceptible to infection and disease. This review seeks to outline the major impairments in vaccine-induced responses due to aging that may be targeted for improvement with design and delivery components encompassing mRNA and DNA vaccine formulations. Results of pre-clinical and clinical applications of these vaccines in aged animal models and humans will also be evaluated to outline current successes and limitations observed in these platforms.

## Introduction

Traditional vaccine platforms, including whole virus, viral vector, and protein-based vaccines have been used for numerous infectious diseases and cancers. Vaccine development is vital globally for preventing infectious diseases and mitigating the risk and economic burden of outbreaks. Recent estimates suggest that bringing a novel vaccine to market in the United States costs approximately $886.8 million and requires 10–15 years of laboratory research, highlighting the extensive and expensive nature of vaccine development (1). Beyond preventative vaccines, therapeutic vaccines for autoimmune diseases and cancers have also been of significant interest. Technological advancement has played a major role in shaping the history of the vaccine development pipeline, leading to the most recent progression of next-generation vaccine platforms. Over the past 20–30 years, nucleic acid-based vaccines (DNA and mRNA) have gained substantial attention. Since their inception, nucleic acid vaccines have evolved significantly. The emergence of DNA vaccines began in the 1990s when investigators discovered injection of plasmid DNA had immunostimulatory properties, subsequently leading to the first DNA vaccine clinical trials (2). Although early preclinical results held promise, clinical trials revealed limited efficacy due to weak immune stimulation and delivery, thereafter sparking advancements in delivery technologies including electroporation and lipid nanoparticles to improve performance. In parallel, beginning in the late 1980s, landmark experiments by Robert Malone served as the foundation for the use of mRNA as a potential drug target (3). From this point, massive developments have been made on the lipid nanoparticle technology necessary to deliver mRNA transcripts. Importantly, the COVID-19 pandemic showcased the rapid development and promising efficacy of mRNA vaccines in humans through its accelerated development. Nucleic acid vaccines offer several potential advantages over traditional vaccine platforms, including rapid and cost-effective production, high antigen specificity compared to whole-organism vaccines, versatile applications across infectious disease, cancer, and genetic disorders, and potent induction of both humoral and cellular responses. Nonetheless, as with any new therapeutic, further investigation into the safety and efficacy of nucleic acid vaccines is still necessary.

While vaccines are essential for the general population to protect against emerging diseases and reduce their spread, the induction of strong vaccine-induced responses in at-risk populations is of paramount interest. Notably, elderly individuals (65+ years) constitute the largest at-risk group, comprising 10% of the world population in 2024, with this percentage expected to more than double by 2050 (4, 5). Given the significant age-related decline in immune responses and the rapidly growing elderly population, enhancing vaccine-induced immunity is crucial. For example, in 2022, mortalities for those 65 and older reached almost 2.5 million with COVID-19 and cancer as two of the top three causes of death (6). This data emphasizes the burden of infectious diseases and cancer on elderly mortality rates and, therefore, the necessity to progress and enhance vaccination strategies in aging populations.

This review will outline age-related impairments in immune cells associated with vaccination responses. Considering the growing efforts in investigating next-generation nucleic acid vaccine formulations, this review will also explore the mechanisms, design, delivery, advantages, and disadvantages of mRNA and DNA vaccines to further our understanding of these platforms. Pre-clinical and clinical studies emphasizing responses elicited in aged animal models and humans will be discussed to highlight the potential of these platforms for vaccinating the elderly and their shortcomings. Finally, as adjuvants and immune modulators are critical components for enhancing vaccine-induced responses in the elderly, previously investigated adjuvants and adjuvant-like properties of nucleic acid vaccines will be examined.

## Immunosenescence and challenges of vaccination in the elderly

**Figure 1:**
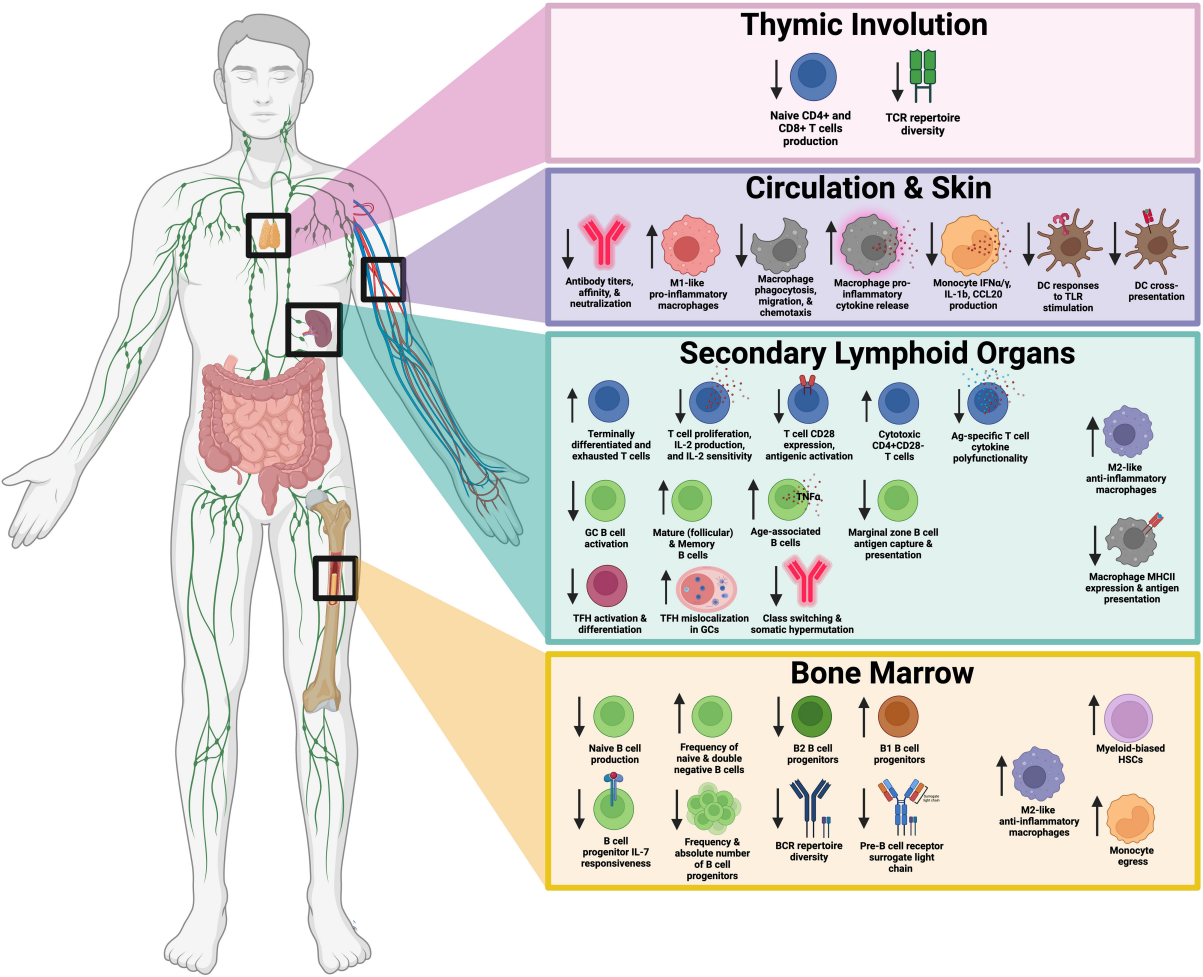
Summary of age-related impairments in T cells, B cells, and antigen presenting cells responsible for vaccine-mediated responses. This schematic illustrates key changes in immune function with age, broken down into four primary regions: the thymus, circulation and skin, secondary lymphoid organs, and bone marrow. Thymic Involution (top panel) leads to decreased production of naïve CD4+ and CD8+ T cells and reduced T cell receptor (TCR) repertoire diversity. In circulation and skin (second panel), altered immune cell populations and function, including changes in antibody titers, macrophage pro-inflammatory cytokine release, and macrophage polarization are observed. Secondary lymphoid organs (third panel), including the spleen and lymph nodes, exhibit changes in T cell subsets, B cell function, and antigen-presentation. Bone marrow (bottom panel) experiences shifts in hematopoietic stem cell populations, reduced B cell and T cell progenitors, and altered macrophage signaling. Downward arrows indicate decreased function or numbers, while upward arrows indicate increased activity or numbers. Created in BioRender.

Aging is a biological process that causes a gradual deterioration in the function of multiple systems of the body, including the immune system. This process of impaired immune function with aging is also known as immunosenescence. With an increase in life expectancy of humans over the past century, the consequential growth of elderly populations has resulted in higher rates of age-related diseases such as cancer in addition to failure of therapeutics for cancers and infectious diseases. Several aspects of immune function at both the innate and adaptive levels decline because of immunosenescence. One key factor influencing immunosenescence is thymic involution, or the progressive reduction in size of the thymus, which acts as a primary lymphoid organ important for T cell maturation. Similarly, aging correlates with an increase in pro-inflammatory status, a phenomenon known as “inflammaging”, representing another hallmark of immunosenescence. Beyond this, aging-mediated hematopoietic stem cell dysfunction, altered T/B cell ratios, impaired antigen responses, accumulation of senescent cells, mitochondrial dysfunction, and genomic instability also underly immunosenescence. This section will focus on age-associated changes to innate antigen-presenting cells (APCs) and adaptive T and B cell function as it relates to vaccine responses (**Figure 1**).

### Immunosenescence associated with antigen presenting cell function

Modern vaccines function by prompting immunological memory through the induction of innate (macrophages, dendritic cells) and adaptive (T, B cells) arms of the immune system by introducing antigen. The development of immunologic memory is initially mediated by innate cells that activate T and B lymphocytes through antigen presentation and the secretion of cytokines. This process enables the differentiation of antigen-specific lymphocytes into memory T and B cells that can be stimulated rapidly upon a subsequent encounter with the same antigen.

The three professional APCs—macrophages, dendritic cells, and B cells—play a crucial role in initiating antigen-specific immune responses and are susceptible to immunosenescence. First, age-related dysregulation of macrophage populations is one of the many alterations responsible for impaired immune function. Generally, macrophages can be grouped into two major types, including M1 (pro-inflammatory) and M2 (anti-inflammatory). In mice, aging is associated with increased M1-like macrophage populations in hepatic and adipose tissues, while M2-like macrophage populations expand in the bone marrow, lymphoid tissues, and muscle, among others (7–9). Furthermore, aged human macrophages were found to exhibit decreased antigen presentation because of lower co-receptor and MHCII expression (10–12). A recent study further identified age-related decreases in the transcription factors MYC and USF1 correlates with reduced phagocytosis, migration, and chemotaxis observed in aged human and murine macrophages (13). With decreased antigen presentation, the induction of antigen-specific adaptive immune responses, particularly through T cells, is impaired in aged individuals, resulting in poor acquired immunity from vaccination and infection. Although elevated M2-like macrophages and decreased antigen presentation are observed with aging, non-specific release of pro-inflammatory cytokines from aged macrophages is commonly attributed to the persistent low-grade inflammation, or inflammaging, seen in aged individuals (14). This increased basal inflammation is likely, in part, due to the ineffective clearance of pathogens by macrophages, resulting in persistent activation. Increased basal inflammation in aged macrophages can further promote T cell immunosenescence, which will be discussed later in this review. Targeting macrophage phagocytosis, antigen presentation, and trafficking through novel optimized vaccine antigens and adjuvants could offer promising approaches to improve vaccine responses in the elderly.

Monocytes are precursors to both macrophages and dendritic cells (DCs). Monocytes have been shown to exhibit weakened expression of notable cytokines including IFNα/γ, IL-1β, and CCL20 with advanced age (15). Although monocytes exhibit reduced pro-inflammatory cytokine secretion, increased egress of monocytes from the bone marrow–due to elevated circulating TNFα–can further contribute to inflammaging upon activation by bacterial products (16). Dendritic cells are the second professional APC and exhibit similar age-related changes to monocytes and macrophages. For example, myeloid and plasmacytoid DCs, which are critical for inducing Th1 and CD8+ T cells (via IL-12) and interferon responses to infection, respectively, show diminished TNFα, IL-6, and IL-12 production following TLR stimulation. These age-related changes to DCs have been associated with impaired vaccination responses to influenza (17). Additionally, aged murine DCs exhibit diminished cross-presentation and subsequent CD8+ T cell priming compared to young mice, in part due to increased production of reactive oxygen species (18).

The third class of professional APCs, which will be discussed further, are B cells. While B cells are critical for antibody production in response to infection and vaccination, their APC capabilities assist in T cell-mediated responses. In humans and animals, aging is associated with the expansion of antigen-experienced aged-associated B cells (ABCs) (19). In contrast to macrophages, monocytes, and DCs, ABCs are considered more efficient APCs than follicular B cells and are heavily associated with autoimmunity development (20). On the other hand, marginal zone B cells, which are important APCs for Th1 effector cell differentiation, exhibit defective antigen capture and presentation with increased age, resulting in impaired T cell-independent immune responses (21).

### Immunosenescence associated with T cell function

T cells are essential for adaptive immunity, as evidenced by severe combined immunodeficiency (SCID), where genetic defects impair T cell development, leading to profound immune dysfunction and vulnerability to infections. Without T cells, the immune system is incapable of producing acquired immunity to infectious diseases, resulting in life-threatening infections and eventual death in humans as well as other animal models of SCID. Within the classification of T cells, several subsets with a diverse range of functions exist, including CD4+ Th1, Th2, Th17, Treg, Tfh, CD8+ cytotoxic T cells, and more. Unfortunately, as individuals age, T cells are among the immune cell types highly impacted by immunosenescence with several hallmarks representing key factors of T cell aging. Evidence of T cell immunosenescence has been widely studied in humans and animal models. First, as previously discussed, thymic involution with age leads to a gradual but impactful decrease in T cell production and maturation. Thymic involution results in the production of fewer naïve CD4+ and CD8+ T cells, with the most notable decrease in CD8+ T cells (22). The diversity of the naïve CD4+ T cell repertoire is maintained up to the age of 65 years, at which point it begins to collapse (23), whereas the CD8+ T cell repertoire decline is observed even earlier in the onset of aging (24). Lower T cell receptor (TCR) clonal diversity and accumulation of terminally differentiated T cells exhibiting dysfunction or exhaustion have also been observed as a result (22). Such age-related changes to naïve T cell populations are correlated with a greater risk for severe infections like COVID-19 (25).

Age associated thymic involution reduces the TCR repertoire and leads to accumulation of mature T cells that are susceptible to repeat antigen activation and recurrent stimulation. Loss of proliferative capacity (26), telomerase activity (27), reduced IL-2 production and sensitivity (28–30), decreased CD28 expression (31, 32), and elevated IFNγ production (33) are among the many phenotypes observed in aging CD4+ and CD8+ T cells. One study found that increased IL-2Rα expression and phosphorylated STAT5 in aged CD4+ T cells direct their differentiation into short-lived effector cells. These changes in IL-2Rα expression were linked to diminished HELIOS expression, a transcriptional repressor (34). Furthermore, CD28 is an important costimulatory molecule for T cells that diminishes with age. As a result of reduced CD28 expression, aged T cells exhibit qualities of cellular senescence including poor antigenic activation and response. The accumulation of CD8+CD28- T cells is associated with a reduced overall immune response to pathogens and vaccines in the elderly (35). Moreover, expansion of CD4+CD28- T cells in the elderly, which exhibit natural killer cell and CD8 T cell-like cytotoxic properties (36) elevate IFNγ production, creating a more pro-inflammatory environment. In a study using single-cell RNA sequencing of CD4+ T cells from young and aged mice, an accumulation of exhausted, cytotoxic, and activated regulatory T cells in aged mice was observed (37). In activated regulatory T cells and cytotoxic CD4+ T cells, enhanced regulatory and pro-inflammatory phenotypes were observed, respectively, indicating their contribution to the decline in immune function. Similarly, an elevated ratio of regulatory T cells to effector T cells in the CD4+ compartment occurs with age and has been correlated to poorer Influenza vaccine responses in aged humans (38, 39). Additional post-vaccination analyses in humans indicate a diminished magnitude of antigen-specific CD8+ T cell responses and CD4+ T cell polyfunctionality in aged individuals (40).

Beyond these changes to T cell populations and function, a specific subset of T cells, known as T follicular helper (Tfh) cells, represent a critical cell type altered with age that are necessary for protective humoral responses to infection and vaccination. Increased age correlates with decreased antibody titers, class switching, somatic hypermutation, affinity, and neutralization, all of which correlate with Tfh function, demonstrated in both research and clinical studies (41–44). Tfh cell activation and differentiation into mature GC Tfh declines with age, leading to impaired antigen-specific immune responses (42). A study in aged mice further revealed Tfh upregulation of CXCR4 in aging leads to spatial mislocalization of GC Tfh cells to the dark zone of germinal centers where they cannot properly interact with GC B cells for induction of somatic hypermutation and affinity maturation (45). Likewise, overall GC response magnitude, volume, and number are reported to decline with age in several mouse immunization models (46–48). With these age-related impairments of T cells in mind, additional considerations for T cell-targeted stimulation must be considered in vaccine design for improved responses in elderly populations. As such, next-generation nucleic acid vaccines have shown great potential at inducing potent T-cell responses compared to traditional vaccine platforms and will be discussed further.

### Immunosenescence associated with B cell function

Humoral responses from aging B cells in response to infection and vaccination also decline with age. As a result, aged individuals are often at higher risk for severe disease and lower protection and durability from vaccination. B cell immunosenescence is associated with reduced total antibody production, and poor-quality antibodies demonstrated with reduced neutralization and affinity. This phenomenon is a result of impaired germinal center B cell reactions necessary for somatic hypermutation and affinity maturation (49). Increasing age is further linked to decreased B cell differentiation within the bone marrow as well as increased aberrant production of mature B cells. Aging correlates with a shift in B cell frequencies as naïve B cells are displaced by memory B cells reducing B cell receptor (BCR) repertoire diversity (50–52), which is associated with poor antigen-specific responses to infection and vaccination. Increased frequency of pro-inflammatory B cells and decreased expression of molecules necessary for immunoglobulin class-switching and somatic hypermutation has also been observed (53, 54).

In an aged mice, the generation of conventional B2 B cells, necessary for antigen-specific humoral responses, is diminished, while B1 progenitors are maintained. This is corroborated by the finding that the frequency and absolute number of B cell lineage precursor populations are decreased in aged mice and this is, in part, due to suboptimal IL-7 responsiveness from these progenitors (55). An additional factor responsible for the decrease of B cell progenitors is the accumulation of myeloid-biased hematopoietic stem cell populations in the bone marrow of aged mice and humans (56, 57). At the molecular level, aging has been associated with decreased expression of E2A and PAX5 transcription factors as well as the pre-B cell receptor surrogate light chain necessary for B cell development and humoral responses (58–60).

When considering age-related changes to peripheral B cell subsets, studies have found that aged humans exhibit a decrease in switched memory B cells and an increased frequency of naïve and double-negative B cells (61, 62). Given the importance of long-lived switched memory B cells for timely antibody responses to repeat antigen exposure, it is clear that aging associated reduced frequencies contribute to an elevated risk for severe infections and poor vaccine responses in the elderly. Additionally, an increase in pro-inflammatory TNFα secretion from memory and double-native B cells in aged individuals negatively correlates with B cell function and vaccine-specific antibody responses (61, 63). Aged-associated B cells (ABCs), separate from follicular (FO) and marginal zone (MZ) B cells (64), are considerably higher in aged humans and mice with nearly 50% of splenic B cells being ABCs in 24+ month old mice (65). More specifically, ABCs were found to be refractory to BCR/CD40 stimulation, whereas innate TLR9/7 stimulation combined with BCR signaling induce Ig secretion and cytokine production (64). The same study also found ABCs favor T cell polarization to the pro-inflammatory Th17 subtype which may further promote inflammaging. In aged humans and mice, ABCs inhibit pro-B cell generation due to elevated TNF secretion (66–68). This increase in ABCs coincides with a decline in the FO B cell pool (67), with studies suggesting that the transition of FO B cells to ABCs may contribute to this shift (62). These age-related changes in B cell function highlight the need for targeted strategies to enhance immune responses in the elderly and improve vaccine efficacy.

### Immunosenescence associated with long-lived immunity

Failure of vaccines to induce long-term protective immune responses in the elderly coincides with the cellular immunosenescence described above. Pre-clinical and clinical findings support the observation that aging leads to a more rapid decline in vaccine-induced antibody titers, often requiring more frequent booster doses to reach and maintain protective levels (69, 70). The combination of reduced bone marrow niches, impaired T cell function, and reduced germinal center responses observed in age-related impairments to vaccination play a role in the reduction of long-lived plasma cell (LLPC) production and survival which represent critical mediators of long-term immunity (71). Therefore, advancements in nucleic acid vaccine technology and adjuvants to target these compartments could offer significant improvement in LLPC production and overall protection induced by fewer doses in the elderly. Critical analysis of these long-term vaccine responses in the context of aging are necessary to progress the development of optimized vaccine formulations.

As shown in **Figure 1**, key changes in immune function associated with age can be broken down into four primary regions: the thymus, circulation and skin, secondary lymphoid organs, and bone marrow. Thymic Involution (top panel) leads to decreased production of naïve CD4+ and CD8+ T cells and reduced TCR repertoire diversity. Circulation & Skin (second panel) exhibit altered immune cell populations and function, including changes in antibody titers, macrophage pro-inflammatory cytokine release, and macrophage polarization. Secondary lymphoid organs (third panel), including the spleen and lymph nodes, exhibit changes in T cell subsets, B cell function, and APC activity. The Bone Marrow (bottom panel) experiences shifts in hematopoietic stem cell populations, reduced B cell and T cell progenitors, and altered macrophage signaling. Taken together, age-related impairments in T cells, B cells, and APCs play a role in reduced immunity to vaccination.

## Nucleic acid vaccines

**Figure 2:**
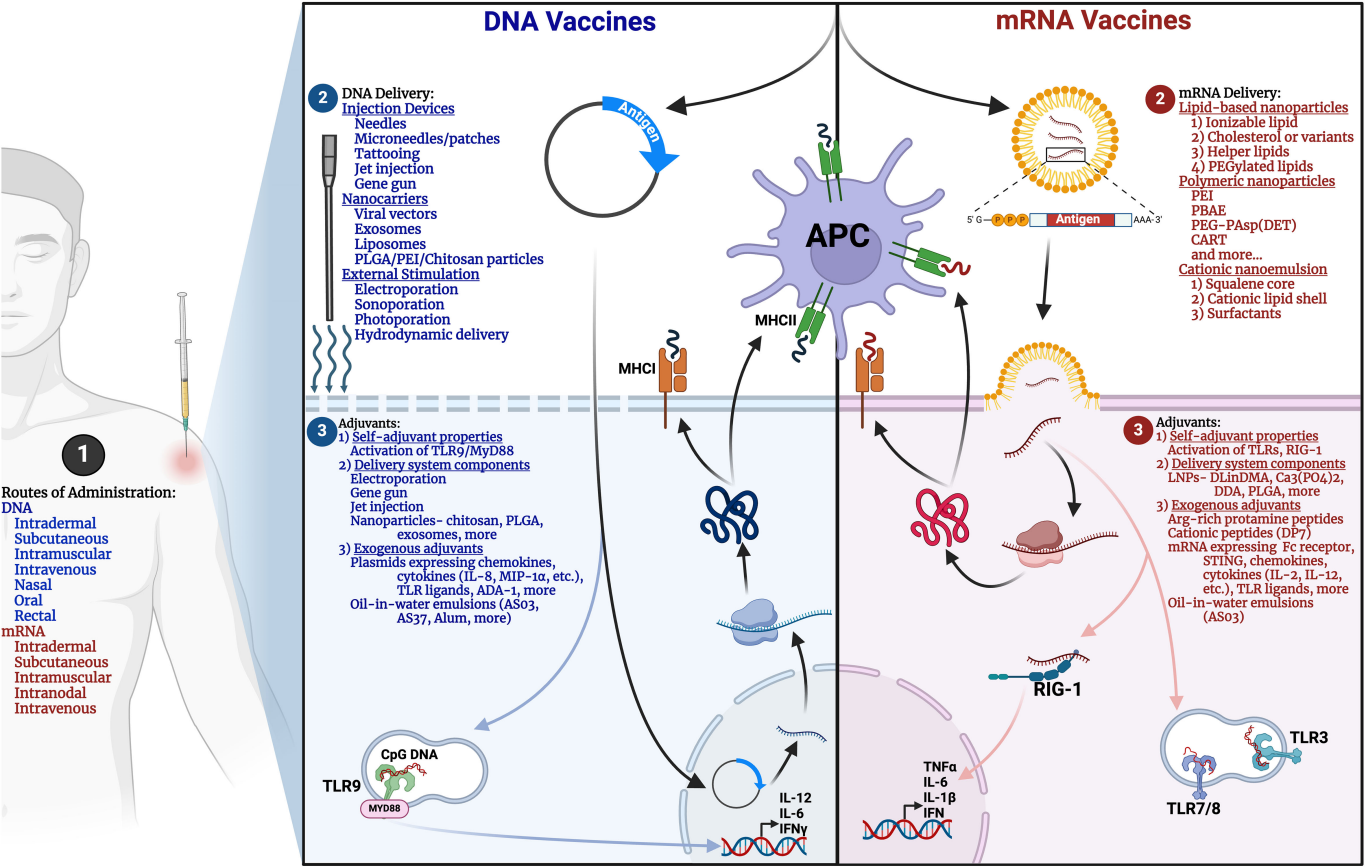
Comparative overview of mRNA and DNA vaccine design. Methods for (1) Routes of administration, (2) Delivery, and (3) Adjuvants for mRNA (right panel, red) and DNA (left panel, blue) vaccines are illustrated. APC, antigen-presenting cell; MHC, Major histocompatibility complex; PLGA, Poly(lactic-co-glycolic acid); PEI, Polyethylenimine; PBAE, poly(β-amino ester); CART, charge-altering releasable transporters; DLinDMA, 1,2-dilinoleyloxy-N,N-dimethyl-3-aminopropane; DDA, dimethyl-dioctadecyl ammonium; ADA-1, Adenosine deaminase-1. Created in BioRender.

Nucleic acid vaccines offer unique properties differentiating them from first- and second-generation vaccine platforms. For example, unlike protein-based vaccines, nucleic acid vaccines introduce mRNA or DNA transcripts that guide cells to produce a protein of interest for subsequent immune responses. In contrast to viral vector vaccines, the genetic material delivered is not accompanied by replication machinery. Furthermore, compared to inactivated virus vaccines which risk virulence reversion, nucleic acid vaccines are safe for administration to immunocompromised individuals. Lastly, across all vaccine platforms, nucleic acid vaccines offer the most rapid production and scalability. This section will cover the mechanism, design, and delivery mRNA and DNA vaccines (**Figure 2** **),** highlighting their advantages, disadvantages, and relevant pre-clinical and clinical studies in aging.

### mRNA vaccine mechanism and design

mRNA is a single-stranded molecule created during the process of transcription from a DNA template and is responsible for the subsequent translation of encoded proteins. Depending on the encoded protein(s), signal sequences can direct the protein to remain within the cell, traffic to the cellular membrane, or be secreted. Over 30 years ago, the ability of mRNA to be administered to produce vaccine antigens rapidly and cost-effectively was envisioned. Compared to other vaccine platforms such as viral vectors and DNA vaccines, mRNA vaccines circumvent potential risks of genome integration and overall exhibit favorable safety profiles (72, 73). Furthermore, mRNA can encode multiple antigens, allowing for vaccination against multiple pathogens and variants. When administered, mRNA will enter the cytoplasm of cells where it can be directly translated into antigenic proteins, enabling a cascade of subsequent innate and adaptive immune responses. This is an advantage compared to DNA vaccines that require access to the nucleus, and require the additional step of transcription, before effective translation of protein antigen. To bring mRNA vaccines to fruition, several considerations must be made for successful administration and induction of immune responses.

In terms of vector design, synthetic mRNA molecules encoding one or multiple proteins can be designed and produced rapidly in a cell-free setting (74). Similar to endogenous mRNA, synthetic mRNA is comprised of a 3’ UTR, poly(A) tail, antigen-encoding region, 5’ UTR, and 5’ cap. On the 5’ end, the 2’-O-methylation must be retained to prevent detection and innate responses by mammalian cytosolic sensors of RNA. Further changes can be made to the 5’ and 3’ UTRs, including the removal of micro-RNA binding sites and AU-rich sites to delay degradation. Additionally, the protein-encoding region of synthetic mRNA can be optimized by incorporating more common codons to enhance protein production. N1-methylpseudouridine, pseudouridine, and other modified nucleosides are also used to optimize protein translation and prevent immune recognition (74).

The delivery of mRNA vaccines has been widely investigated since the 1990s but were first broadly deployed in humans with the release of the SARS-CoV-2 spike mRNA vaccine. To deliver mRNA, it must breach the barrier of cellular membranes to enter the cytosol where it can be translated to protein. Due to the negatively charged membrane, negatively charged mRNA must be encapsulated to avoid repulsion. Three main nanoparticle delivery systems have been used in mRNA vaccine design, including lipid-based nanoparticles (LNPs), polymeric nanoparticles, and cationic nanoemulsion. Besides encapsulating the mRNA within their core, these delivery systems also share cationic or ionizable molecules. In the case of LNPs, these nanoparticles often contain four major components, including ionizable lipids (DLin-MC3-DMA (75), SM-102 (76), A6 (77), and more), cholesterol or cholesterol variants (β-sitosterol (78) and 20α-hydroxycholesterol (79)), helper lipids (DSPC (80) and DOPE (81)), and PEGylated lipids (ALC-0159 (82) and PEG-DMG (82)). The cationic lipid facilitates encapsulation of the negatively charged mRNA but may induce toxic pro-inflammatory responses (83). The addition of an ionizable lipid can improve the safety profile, extend circulation time (84), and promote endosomal release of the mRNA (85). Investigation into other ionizable lipids for mRNA delivery has expanded rapidly and includes lipids targeting immune cells such as DCs (86) and T cells (87) to improve immunogenicity. Furthermore, inclusion of cholesterol or its derivatives improves LNP stability and endosomal fusion (86), whereas helper lipids influence fluidity, endosomal fusion (88), and can enable organ specificity (89). Finally, PEGylated lipids can be used to alter the size, circulation time, and efficacy of LNPs (90, 91).

Polymeric nanoparticles like PEI (92), PBAE (93), PEG-PAsp(DET) (94), and charge-altering releasable transporters (CART) (95) can be used alternatively to form condensed polymer-mRNA complexes for effective mRNA delivery. Similar to LNPs, different polymers can be chosen to influence organ specificity (96), pH responsiveness (97), and more. Modifications such as the introduction of disulfide linkages and PEGylation can further modulate toxicity (98) and organ specificity (94, 99–101). Lastly, cationic nanoemulsions have also been studied for mRNA delivery. This delivery system contains a squalene core, a cationic lipid shell, and surfactants like Tween 80 for the electrostatic binding and adsorption of mRNA (102). Investigation into peptide-based nanocomplexes for mRNA delivery are also under investigation and has shown promising results for eliciting T-cell mediated immunity (103). The continued advancement of mRNA vaccine technology presents exciting opportunities for improving immune responses in aging populations, with potential strategies focusing on optimizing delivery systems and enhancing antigen-specific immunity to address age-related immune decline.

### Pre-clinical testing of mRNA vaccines related to aging

mRNA vaccines are being developed for a broad range of infectious diseases (e.g., Influenza, *Clostridioides difficile*, Norovirus, Tuberculosis, Herpes Simplex Virus, Hepatitis C), genetic disorders, and cancers, that disproportionately affect elderly individuals, however there is further research necessary to evaluate their efficacy pre-clinically, in aged models (104). To review our current understanding of mRNA vaccine efficacy in aged individuals, we investigated the most up-to-date preclinical studies using aged models for COVID-19 and Influenza A vaccination. Evaluating the efficacy of mRNA vaccines in elderly populations requires a critical analysis of overall protection, humoral immune responses, and cellular immune responses.

The use of aged mouse models has proven to be a useful tool to further our understanding of the impact of aging on the immune response and level of protection conferred by mRNA vaccines specifically in the context of COVID-19 mRNA vaccines. Brooke et al., combined an aged mouse model with human clinical data to characterize an impaired Th1 response to the COVID-19 vaccine *in vivo* (105). Chen et al. utilized a similar aged model to conduct rechallenge experiments with COVID-19 to better characterize breakthrough infection, finding that even with 2 doses of the vaccine, aged mice were more susceptible to infection (106). Taken together, these *in vivo* models allow us to identify additional opportunities for optimized treatments and prevention strategies against SARS-CoV-2 among older individuals (107). Similarly, preclinical studies investigated the efficacy of mRNA vaccines for Influenza A in aged models. Specifically, one group has developed an mRNA vaccine capable of inducing long-lived protective immunity to Influenza A in very young and very old mice, as evidenced by the effective humoral and cellular responses elicited (108). These findings highlight the critical role of aged models in advancing the development and optimization of mRNA vaccines, providing valuable insights and opportunities for improving vaccine efficacy and durability in older populations across a range of infectious diseases.

### Clinical testing of mRNA vaccines related to aging

A variety of mRNA vaccines are currently undergoing clinical trials targeting infectious diseases beyond COVID-19, including Human immunodeficiency virus (HIV), Zika virus, Nipah virus, and Respiratory syncytial virus (RSV)—as well as genetic disorders and various cancers (109), however there are limited trials that look at the efficacy of these mRNA vaccines specifically in aged populations. While randomized clinical trials typically exclude elderly populations, the heightened vulnerability of this group to COVID-19 led to their inclusion in licensing trials under the exceptional circumstances of the pandemic which allowed for considerable insight into the efficacy of mRNA vaccines in elderly (109). Here, we will review the key findings from clinical trials evaluating the efficacy of mRNA vaccines in aged populations, focusing on their response to the COVID-19 pandemic, as well as trials for RSV and Influenza A. While we, among others, have characterized the impact of immunosenescence on vaccine efficacy (110, 111), mRNA vaccines demonstrated surprising efficacy in aged individuals during the COVID-19 pandemic and in multiple clinical trials (112). A large-scale matched case–control study of individuals aged 80–83 showed that emergency hospital admissions were 75.6% lower among those fully vaccinated with BNT162b2 compared to unvaccinated controls, and SARS-CoV-2 positivity was reduced by 70.1% in the vaccinated group. These findings, among others, underscore the high efficacy of COVID-19 vaccine in the elderly, providing strong protection against infection and hospitalization (113, 114).

However, beyond high-level efficacy comparing vaccinated vs. unvaccinated groups, it is important to interrogate differences in the immune response between young and older *vaccinated* adults to form a complete picture of the immune response to mRNA vaccines. Results from previous aging-focused mRNA vaccine clinical trials in response to COVID-19 highlight three critical takeaways (1): the reduced humoral response in aged populations, (2) the importance of repeat dosing to improve the humoral response, and (3) the challenge in driving cellular immunity for aged populations. In the context of the BNT162b2 COVID-19 vaccine (115), the compiled results of many trials demonstrate the importance of repeat dosing to mount a protective humoral response in elderly patients. The first clinical challenge observed was the delayed response/increase in dosing required to mount a protective humoral response. A trial in Greece examining differences in antibody responses after 1 or 2 doses of the BNT162b2 COVID-19 vaccine (115) found that individuals over 85 exhibit a 41.18-fold increase in neutralizing antibodies after the second dose. Furthermore, in the cohort of 400 individuals who received the first dose, the mean antibody response reached 69.75%, whereas among the 297 recipients of the second dose, it increased to 98.99% (116). This was further demonstrated by Collier, et al. when investigating immune responses in elderly individuals against key variants of concern (VOCs) (117). After the first dose, older individuals, particularly those over 80, exhibited reduced serum neutralization, IgG, and IgA levels, with lower neutralization potency against Alpha, Beta, and Gamma variants compared to the wild-type virus. Many individuals over 80 lacked neutralizations against variants of concern (VOCs). However, the second dose restored neutralization against VOCs across all age groups. Elderly responders also showed reduced somatic hypermutation in class-switched cells compared to younger individuals (44). Interestingly, Jergoiv et al. highlighted that while there was robust humoral immunity achieved in the older cohort, it was delayed in onset. More specifically, the older cohort displayed lower neutralizing capacity at 7–14 days following the second dose that equilibrates with the younger cohort after 2–3 months (118). Despite the increase in humoral response after repeat dosing, a decrease in overall durability in aged individuals was displayed by Korosec et al., finding that individuals aged 18–55 are predicted to have a four-fold advantage in humoral response compared to those aged 56–70 and 70+ by 8 months following two doses (119). Taken together, these data highlight that the humoral response in elderly groups in response to the COVID-19 vaccine is delayed and requires additional dosing.

To investigate if increased dosing could close the disparity gap observed between aged and young populations, some groups demonstrated that there was a rescue of the humoral immune response in the elderly after 3–4 doses of the COVID-19 vaccine. To complement the increasing immunity that is conferred with 2 doses of the vaccine, Renia et al. found that older individuals take longer to achieve vaccine-induced immunity but maintain more sustained responses at 6 months. A third dose significantly enhances antibody levels in older adults against the Wuhan strain and, Delta and Omicron variants (120). Shapiro et al. also looked at dose response across various age groups and found that the third dose of vaccine restored functional antibody responses and eliminated disparities caused by sex, age, and frailty in older adults (121). Furthermore, 4 doses of the vaccine enhanced the neutralizing antibodies against the Wuhan Strain and Omicron (122).

T cells are critical in driving the adaptive immune response of mRNA vaccines, as previously described, cellular immunity is impaired by age-mediated reduced thymic activity (123, 124). The composition and status of both naive and memory T cell repertoires are critical in determining the quality of immune responses, including those to SARS-CoV-2 (125). While some groups found that after repeat vaccination, older vaccinees manifest cellular immunity comparable to the younger individuals against early-pandemic SARS-CoV-2 and more recent variants (44, 118, 119), a more comprehensive study that included longitudinal analysis of TCR tracking in conjunction with pre/post-vaccination CD4+T cell analysis argues that it might be more complex (125). Saggau et al. found that the SARS-CoV-2-specific T cell repertoire determines the quality of the immune response to vaccination. They investigated both naïve and memory T cell compartments and found that the T cell expansion in both compartments in response to the mRNA vaccine was severely compromised—calling for a need for alternative strategies to increase the T cell response to mRNA vaccines in the elderly (125). These findings underscore the pivotal role of CD4+ T cells in driving mRNA vaccine responses and highlight the need for alternative strategies to enhance T cell responses in older individuals, given the compromised expansion of both naive and memory T cell compartments with aging.

In addition to insights gained from the COVID-19 vaccine rollout, five completed and five ongoing clinical trials are testing mRNA vaccines for influenza and RSV in older individuals (A list of completed and ongoing clinical trials of mRNA and DNA vaccines involving aged individuals, outside of those tested for COVID-19 is shown in **Table 1**). Most compelling is a Phase II-III clinical trial with an mRNA vaccine targeting RSV that enrolled older adults over 60, showing high efficacy against RSV-associated lower respiratory tract disease (83.7% with two symptoms and 82.4% with three symptoms) and 68.4% efficacy against acute respiratory disease. The vaccine also protected both RSV A and B. There is additional early promise from 2 Phase I clinical trials that showed increased humoral and cellular responses in aged and young adult groups (126, 127). These findings highlight the potential of mRNA vaccines to significantly improve protection against respiratory infections in older adults, warranting further investigation and development. A significant area for opportunity for future mRNA vaccine development for the elderly is in cancer applications. mRNA vaccines allow for greater precision and specificity in delivering tumor associated antigens at a personalized level, while maintaining effective scalability and low cost. Beyond these advantages, mRNA vaccines are shown to elicit robust T cell responses in younger demographics: making this an optimal platform to consider (128). With the broad increase in the aged population and associated increased incidence of cancer in elderly, mRNA vaccines for cancer must be investigated clinically for elderly individuals.

**Table 1:** List of completed and ongoing clinical trials of mRNA and DNA vaccines involving aged individuals.

NCT Number:	Conditions:	Sponsor:	Phases:
mRNA Vaccine Trials			
NCT05829356	Influenza	Sanofi Pasteur, a Sanofi Company	PHASE1
NCT05446740	Influenza	GlaxoSmithKline	PHASE1
NCT05426174	Influenza	Sanofi Pasteur, a Sanofi Company	PHASE1
NCT05252338	Influenza	CureVac	PHASE1
NCT04528719	Respiratory Syncytial Virus	ModernaTX, Inc.	PHASE1
NCT06431607	Influenza	GlaxoSmithKline	PHASE2
NCT06374394	Respiratory Syncytial Virus	GlaxoSmithKline	PHASE3
NCT06237296	Respiratory Syncytial Virus and/or Metapneumovirus	Sanofi Pasteur, a Sanofi Company	PHASE1
NCT06125691	Influenza	Arcturus Therapeutics, Inc.	PHASE1
NCT05823974	Influenza	GlaxoSmithKline	PHASE1/PHASE2
DNA Vaccine Trials			
NCT04336410	SARS-CoV-2	lnovio Pharmaceuticals	PHASE1
NCT04915989	SARS-CoV-2	Genexine, Inc.	PHASE1
NCT04742842	SARS-CoV-2	University of Sydney	PHASE1
NCT00300417	West Nile Virus	NIAID	PHASE1
NCT01587131	Influenza	University of Manitoba	PHASE1
NCT05163223	HER2 Low Breast Cancer	Aston Sci. Inc.	PHASE2
NCT00436254	HER-2 Positive Stage III-IV Breast Cancer or Ovarian Cancer	University of Washington	PHASE1
NCT05904054	SARS-CoV-2	lmmuno Cure 3 Limited	PHASE2

### Advantages and disadvantages of mRNA vaccines

mRNA vaccines represent a significant advancement in immunization technology, offering several advantages for the elderly. These advantages include rapid development, high efficacy, and a favorable safety profile. The rapid, cost-effective, and scalable production of mRNA vaccines enables swift responses to emerging health threats that disproportionately affect older adults. The efficacy of mRNA vaccines in preventing severe disease outcomes in older populations was demonstrated during the COVID-19 pandemic (112–114). mRNA have improved safety implications for elderly because it is non-infectious compared to viral vector or live attenuated vaccines which potentiate additional risks in aged individuals. Furthermore, the transient expression of mRNA that results from its single stranded nature, may be an additional safety feature for vaccines in the context of targeting aged populations and its application in cancer therapy. While additional clinical trials are needed to consider the safety profile of mRNA vaccines in elderly populations, the previously described clinical trials for mRNA-based RSV vaccines found no evident safety concern (126). Broadly, mRNA vaccines have advantages over alternative traditional approaches such as DNA plasmids, viral vector vaccines or live attenuated vaccines. Compared to DNA vaccines, mRNA vaccines do not require access to the nucleus and does not pose the threat of chromosomal integration (129). Compared to traditional viral vector or live attenuated vaccines mRNA vaccines can be rapidly designed, scaled quickly and at very low cost.

However, like any emerging advancement in medicine, there are existing challenges, limitations and opportunities for improvement for the mRNA vaccine platform for elderly patients. Key limitations of mRNA vaccines for elderly populations include stability, durability and potential adverse immune reactions. First, as mRNA acts as a transient intermediate between DNA and protein, its short half-life in the body is of primary concern. mRNA can be broken down rapidly by deadenylases, the exosome, ribonuclease, and endonucleases, so carrier design is critical to mRNA efficacy. These concerns are also critical for storage and transportation, particularly for countries without access to rapid transportation and devices necessary to proper storage. Second, improving the durability and memory of immune responses to mRNA vaccines is an area that must be further studied in aged individuals. While mRNA vaccine mediated immune response can improved with increased dosing in aged individuals (120–122), there is a lack of clinical evidence showing long lived memory to mRNA vaccines in aging. Increased dosing is a potential strategy to improve durability in elderly, however it could also increases the risk associated with off target, adverse immune reactions for elderly. Many steps have been taken to improve the safety of mRNA, to mitigate adverse immune reactions, such as the 2’-O-methylation strategy to mRNA which prevents detection by innate pattern recognition receptors (PRRs). However, the concern mRNA vaccination and development of autoimmune disease is a concern for elderly. In the context of SARS-COV2, it was concluded that mRNA vaccines were not associated with an increased risk of most autoimmune connective tissue diseases (AI-CTDs) (130), however, understanding long term safety profile and risk associated with mRNA vaccines in elderly requires further investigation. Overall, further advancement and characterization of mRNA vaccine stabilization, potency and durability is necessary, particularly for purposes of distribution and application to individuals with impaired vaccine responses like the elderly.

### DNA vaccine mechanism and design

DNA-based vaccines are a third-generation vaccine platform that relies on the use of artificially synthesized plasmids encoding an antigen of interest to be transfected within cells of the body for subsequent production of antigen *in vivo.* Beginning in the 1990s, it was found that intramuscular injection of plasmid DNA could be performed to induce protein expression and that most of the DNA could be taken up into the nucleus of cells without specialized delivery methods (131). Since this study, investigations of genetically engineered DNA plasmids for vaccine use have expanded, showing the scalable and stable nature of this platform. DNA vaccines are designed to encode an antigen of interest, ranging from cancer-specific molecules to proteins from infectious organisms, that can subsequently induce antigen-specific immune responses. To effectively induce transcription and translation of a DNA vaccine, a delivery system is used to enable the plasmid to enter the nucleus of cells within the body.

Several routes of administration and delivery systems have been investigated over the past several decades. DNA vaccines can be delivered through the skin (intradermal, intramuscular, intravenous, subcutaneous), nasally, orally, or rectally. Depending on the administration method used, injection devices, nanocarriers, or external stimulation can be applied to facilitate DNA delivery to cells. In terms of injection devices, traditional needles and microneedles enable penetration of the epidermis. Microneedles prepared as adhesive patches facilitate delivery to the interstitial fluid. This delivery method was shown to improve DNA vaccine immunogenicity in an influenza model by inducing potent humoral and cellular responses compared to traditional intramuscular injection (132). Tattooing represents a unique method for intradermal vaccine delivery by introducing numerous skin perforations in seconds with controlled vaccine release over a larger area than traditional needles or micropatches. Improved tattooing methods in Ebola and HIV DNA vaccine models show superior T and B cell responses compared to intradermal and intramuscular delivery across several animal species, including mice, rabbits, and non-human primates (133). Needle-free methods for DNA vaccine delivery have also been explored. Jet injection, such as the Biojector (134), is an injection device that forces microdroplets carrying the vaccine of choice into the skin using high pressure for subcutaneous, intradermal, or intramuscular delivery (135). Separately, the gene gun enables gold microparticles to be coated with a DNA vaccine and delivered directly into intradermal or mucosal cells with compressed helium gas (136). In an HPV DNA vaccine model, the gene gun was shown to induce potent CD8+ T cell responses comparable to electroporation (137).

A variety of nanocarriers have been evaluated for DNA vaccine delivery. Nanocarriers are capable of improving antigen delivery by protecting mRNA from degradation and promoting cellular uptake and antigen presentation. They can also be prepared with innate-stimulating properties such as TLR agonists and designed to induce specific cytokine responses. Such carriers include viral vectors (138), exosomes (139), liposomes (140), PLGA (141), PEI (142), and chitosan (143) particles. While each nanocarrier offers different benefits for DNA vaccine delivery, collectively, they enable the protection of DNA plasmids from degradation, increase circulation stability, and offer targeted tissue delivery (136). Finally, external stimulation represents a commonly used form of DNA vaccine delivery. Electroporation applies electrical impulses to transiently permeabilize cellular and nuclear membranes to deliver DNA plasmids to the nucleus of cells for transcription and translation. This method is common for intramuscular DNA vaccination, allowing for versatile delivery of naked DNA or nanocarriers in addition to self-adjuvating properties by inducing minor inflammation and trafficking of APCs and lymphocytes to the site of vaccination (144). Similarly, sonoporation (145), photoporation (146), and hydrodynamic delivery (147) of DNA vaccines use ultrasound waves, lasers, and capillary pressure, respectively, to create small pores in cellular membranes for DNA entry. Although many delivery methods exist for DNA vaccination, the cancer or infectious agent of interest must be considered before vaccine design to ensure optimal immune responses are achieved.

### Pre-clinical testing of DNA vaccines related to aging

While several pre-clinical studies of DNA vaccines exist, examination of these vaccines in aging models can help provide evidence for the potential of this platform’s immunostimulatory capabilities and areas for improvement. Few pre-clinical DNA vaccines have been tested in models of aging, possibly in part due to the expense of aged animals. However, those that exist improve our understanding of the benefits and shortcomings of DNA vaccine formulations and, therefore, will be discussed for this review. In the context of infectious diseases, DNA vaccines have shown promising results in aged models. Starting with a current and clinically relevant viral target, the recent COVID-19 pandemic sparked interest in DNA vaccines targeting SARS-CoV-2, which has proven particularly threatening to the aged population (148). In one study of a DNA vaccine encoding chimeric SARS-CoV-2 S1 spike protein fused to a trimerization transmembrane region, aged mice were vaccinated in a two-dose regimen for subsequent analysis of antigen-specific adaptive immune responses (149). Cui et al. found two doses induced strong humoral responses with neutralizing capacity to both Wuhan and Delta spike variants, while a third booster dose after 6 months significantly boosted the magnitude of these responses in aged C57BL/6 mice like young mice. Similarly, spike-specific T cell TNFα responses were induced in aged mice, although to a lesser extent compared to young. In a separate study, DNA vaccines against Influenza A haemagglutinin (HA) and nucleoprotein (NP), another clinically relevant viral pathogen disproportionately impacting elderly populations, were tested in young and aged BALB/c mice (150). Both HA- and NP-DNA plasmids induced antibody responses in aged mice, though to a lesser extent than observed in young mice. Notably, the NP-DNA vaccine induced similar cytotoxic CD8+ T lymphocyte activity in both age groups and protected against low-dose intranasal challenge based on weight loss recovery. However, aged mice were not protected against high-dose A/HR/68 (H3N2) challenge, suggesting vaccine alterations to target improved antibody neutralization capacity in aged mice is necessary. In contrast, investigation of a DNA vaccine targeting the thrombocytopenia syndrome virus (SFTSV) showed that aged ferrets developed strong T cell and antibody responses to SFTSV and were completely protected from lethal challenge (151). Beyond these viral targets, aged models of DNA vaccination have also been tested in the context of malaria. Using a plasmid encoding the circumsporozoite protein of the Plasmodiumyoelii malaria parasite, Klinman et al. found vaccinated aged BALB/c mice exhibited lower humoral and CD8+ T cell responses compared to young mice, with an overall 40% protection from challenge compared to 80% in young mice (152). It should be noted that the aged mice used in this study were of extreme age (26 months compared to standard 18–24 months used for aged models), but these findings, along with the previously described viral DNA vaccine models, indicate improvements can be made to induce greater immunogenicity in aged individuals.

Besides infectious diseases, cancer, autoimmune disorders, and neurodegenerative diseases represent attractive targets for DNA vaccines, particularly in the context of aging. For example, breast cancer is one of many cancers that are significantly more common with increased age. In an attempt to target a tumor antigen commonly detected in metastatic breast cancer, young and aged mice were vaccinated with a plasmid encoding Mage-b intramuscularly followed by challenge with mild (4TO7cg) and aggressive (4T1) syngeneic metastatic mouse breast tumor models (153). Aged mice exhibited lower IL-2 and IFNγ levels in the draining lymph nodes and spleen, along with a reduced frequency of Mage-b-specific CD8+ T cells, compared to young mice. These immunological deficits correlated with a diminished protective response against tumor challenge. These results suggest that there is room for improvement, and tailoring DNA vaccination to improve cytokine and CD8+ T cell responses could enhance cancer vaccination efficacy in aged models. For example, electroporation following intramuscular delivery can improve immune responses, as demonstrated by a pre-clinical study investigating a DNA vaccine against the HER-2/neu antigen expressed in many breast tumors and adenocarcinomas (154). Compared to intramuscular delivery alone, electroporation significantly improved humoral and cellular responses to HER-2/neu and induced complete protection (40% increase) against HER-2/neu overexpressing cancer cell line challenge in aged mice. Beyond cancer, neurodegenerative diseases such as Alzheimer’s disease (AD) heavily impact aged individuals. As a result, studies investigating DNA vaccines targeting amyloid-β (Aβ) plaques that accumulate in the brain of Alzheimer’s patients have been of interest. In one study, DNA immunizations with the amino-terminal Aβ (1-11) fragment exposed on the surface of HBsAg particles resulted in high anti-Aβ antibody titers, reduced Aβ plaques, and reduced cognitive impairments in an aged transgenic mouse model of AD (155). In a separate study, DNA vaccination against recombinant Aβ3–10 similarly reduced Aβ plaques, synaptic and neuron loss, and memory impairment in aged transgenic mice (156). These studies exemplify the promise of DNA vaccines in aged individuals beyond infectious disease targets.

### Clinical testing of DNA vaccines related to aging

While pre-clinical evaluations of DNA vaccines in aging models are sparse, fewer clinical trials in elderly humans exist (**Table 1**). In a more recent clinical trial for the SARS-CoV-2 DNA vaccine INO-4800 (NCT04336410), a 2.0 mg dose induced strong and durable humoral responses in all age groups, including those >65 years, although IFNγ ELISpot and CD8+ T cell analyses suggested poorer, but detectable, T cell cytokine responses to vaccination (157). In a promising clinical trial for a West Nile Virus (WNV) DNA vaccine (NCT00300417), subjects aged 18–50 years and 51–65 years were vaccinated against the premembrane protein and the E glycoproteins of the NY99 strain (158). Following a three-dose regimen, the majority of subjects in both age groups exhibited T cell responses, and the older age group demonstrated antibody responses with similar frequency, magnitude, and durability as those observed in the younger participants. With no licensed vaccine against WNV available for use in humans, this trial indicated promising results for eliciting protection against WNV in young and older individuals using next-generation vaccine platforms where traditional platforms have failed. Furthermore, a phase 1 clinical trial evaluating Inovio’s H1N1 Influenza A DNA vaccine in healthy elderly subjects (NCT01587131) found a single dose in conjugation with the seasonal flu vaccine induced protective immune responses in 40% of subjects compared to 20% in those who received the seasonal flu vaccine alone (159). These results indicated the potential for this DNA platform to promote potent, protective immune responses against influenza, particularly for at-risk elderly populations, which current flu vaccines have failed to achieve.

As previously mentioned for pre-clinical vaccines, DNA vaccines for cancer have also reached the clinical trial phases. In a phase 1 trial for a DNA vaccine encoding HER-2/neu for advanced-stage ERBB2-positive breast cancer (NCT00436254), participants aged 34–77 years were immunized three times with one of three dosages (50, 100, or 500ug) (160). Most subjects developed HER-2-postive Th1 T cell responses with minimal toxicity, and the 100ug dose has progressed to phase 2 trials (NCT05163223). While these studies include elderly participants, details on age-dependent variations in vaccine responses were not evaluated. Similarly, although other cancer DNA vaccines are in clinical trials, age-associated responses have not been examined. Further analyses of DNA vaccine immunogenicity and efficacy in aged participants are necessary and could aid in the optimization of vaccine formulations and delivery for the elderly.

### Advantages and disadvantages of DNA vaccines

The DNA vaccine platform offers its own unique set of advantages and disadvantages. Beyond the advantages of low manufacturing costs and rapid production shared between mRNA and DNA vaccines, DNA vaccines offer several additional advantages including stability, safety, flexibility in design, and the ability to induce robust immune responses. The high stability of DNA vaccines makes them convenient to store, transport, and distribute. Furthermore, DNA offers no safety risk of viral transformation like that associated with live attenuated vaccines or other side effects associated with inactivated cell vaccines. In terms of vector design, DNA vaccines are flexible and easily manipulated and offer the potential to encode one or multiple antigens of interest for protection against one or multiple diseases in a single plasmid. Beyond the ability to encode multiple antigens, DNA vaccines enable host cells to produce antigens with the post-translational modifications necessary for the native-like structure (161). Lastly, DNA vaccines can elicit a broad spectrum of immune responses, including humoral and cellular immunity.

Unfortunately, concerns surrounding the use of DNA in vaccine design have prevented widespread development and use clinically. Integration into the host genome is of paramount concern, particularly in the public eye, despite DNA exhibiting a low tendency to integrate when circular in a plasmid (160, 162). Additionally, the amount of DNA necessary for vaccination is small, further lowering risk of integration. Nonetheless, administration of a DNA vaccine, in rare cases, could result in insertional mutagenesis potentially resulting in gene malfunction, inactivation, or even upregulation of gene expression. To help decrease risk for such insertions, the FDA has offered recommendations to increase plasmid DNA supercoiling to greater than 80% and keep the DNA copies administered below 10,000 (163). In addition to DNA integration, another major safety concern of DNA vaccines is the induction of autoimmunity. By injecting foreign DNA encoding an antigen of interest, DNA vaccines have the potential to elicit anti-DNA humoral responses. Although an early animal study suggested DNA immunization induced anti-DNA antibodies in mice (164), other work suggests there is little to no risk for autoimmunity development, particularly when using non-viral delivery methods (165, 166). Other disadvantages also exist. For example, delivery methods such as electroporation can cause discomfort not associated with other vaccine platforms. Further work on DNA vaccine design and delivery, particularly in the field of nanocarriers, may promote the development and distribution of this platform in the future. However, addressing the risk for DNA integration and autoimmunity is paramount for the progression of DNA vaccines to widespread use.

## The role of adjuvants in enhancing nucleic acid vaccine responses

Adjuvants represent a critical component of vaccine formulations necessary for enhancing antigen-specific immune responses. Particularly for individuals with impaired immune functions, such as the elderly, adjuvants are necessary to elicit protective immune responses to vaccination. As the number of vaccine adjuvants has grown, their diversity has expanded to include natural extracts, synthetic compounds, oil-in-water emulsions, and more. The discovery of adjuvants and their application to boost vaccine responses first occurred in the 1920s when the immune-enhancing properties of aluminum salts were observed when co-formulated with vaccine antigens (167). After successfully translating aluminum as an adjuvant to human vaccinations in the 1930s, it remained the only licensed vaccine adjuvant for roughly seven decades. Since then, many new adjuvants have been discovered, and their applications to specific vaccine platforms and pathogen types have been investigated closely. For example, aluminum does not function well for vaccine-induced immune responses to intracellular pathogens (168). Importantly, in the context of nucleic acid-based vaccines, the choice of adjuvants varies. For both mRNA (**Table 2**) and DNA (**Table 3**) vaccines, these adjuvants can be broken down into three overarching categories, including (1) self-adjuvant (2), delivery system components, and (3) exogenous adjuvants.

**Table 2:** List of delivery component and exogenous adjuvants for mRNA vaccine formulations.

mRNA Vaccine Adjuvants		
Delivery Components		
Adjuvant:	Modulatory action	References:
DLinDMA	↑Germinal center Tfh and B cell responses ↑Antibody neutralization	(169–171)
LCP/Calcium Phosphate	↑CD80/86 expression and DC maturation	(172)
DDA	↑Th1 responses	(173)
DOTMA/DOPE	↑NLRP3 activation and IL-1β production	(174)
C1	↑TLR4 and DC activation ↑IL-6 and IL-12p70 production	(175)
A2	↑STING activation ↑CXCL10, IFNγ ↑Antigen-specific CTL responses	(86)
C12-TLRa	↑TLR7/8 activation	(176)
SAL12	↑STING activation and IL-1β production	(177)
Exogenous Adjuvants		
Cationic peptide DP7	↑CD103+ DC maturation and antigen presentation	(178)
Arginine-rich protamine peptides	↑TLR7/8 activation ↑B and T cell-dependent responses	(108, 179)
Active STING	↑Type I IFN production ↑Antigen-specific T cell responses	(180)
R848	↑TLR7/8 activation	(169, 181)
a-GC	↑NKT cell activation ↑Anti-tumoral responses	(182)
IL-12p70	↑Cellular and humoral responses in aged mice	(181, 183)
AS03	↑Antigen-specific Th1/Th2 responses	(184, 185)

**Table 3:** List of delivery component and exogenous adjuvants for DNA vaccine formulations.

DNA Vaccine Adjuvants		
Delivery Components		
Adjuvant:	Modulatory action	References:
Electroporation	↑APC and T cell trafficking to vaccination site ↑TNFα and IL-1β release ↑Response duration	(144, 186–190)
Gene gun	↑Th2-biased responses	(191, 192)
Chitosan	↑Humoral/cellular responses ↑IFNγ production ↑Macrophage activation	(193–196)
PLGA	↑MHCI antigen presentation ↑T cell cytokine production	(197, 198)
PEI	↑T cell activation/proliferation ↑Humoral responses ↑APC activation	(199–202)
Liposomes	↑Humoral/cellular responses	(203–206)
Exosomes	↑DC activation	(207)
Exogenous Adjuvants		
Encoded cytokines and chemokines (IL-2, IL-6, IL-7, IL-8, IL-12, IL-15, GM-CSF, MCP-1, MIP-1α, RANTES, CCL28, CCR10L)	↑T cell activation ↑APC recruitment and activation	(183, 208–219)
Encoded PD-1	↑CD8+ T cell responses	(220, 221)
Encoded adenosine deaminase-1	↑Antibody neutralization ↑T cell cytokine responses ↑Tfh-inducing cytokines	(222–225)
AS03	↑Antigen-specific Th1/Th2 responses	(184)
Alum	↑NLRP3 activation and IL-1β production ↑DC activation ↑Antigen-specific Th2-biased responses	(226)
CpG motifs	↑TLR9 activation	(227)
Monophosphoryl lipid A	↑TLR4 activation ↑Humoral and Th1 responses	(228, 229)
Enterotoxins/Toxin derivatives	↑APC antigen presentation ↑Th1/Th2 cytokine responses	(230–233)

### Self-adjuvant properties

Both mRNA and DNA vaccine platforms can exhibit self-adjuvating properties without the presence of additional mediators. mRNA alone has the capability of acting as an immunostimulatory molecule. The innate immune system is equipped with pattern recognition receptors that can bind foreign or host RNA and elicit responses. For example, TLR3 and TLR7/8 recognize double-stranded and single-stranded unmodified mRNA, respectively. Detection of poly uracil (U) and short double-stranded RNA with 5’ triphosphate can activate RIG-1 and TLR3, leading to the induction of pro-inflammatory cytokines TNFα, IL-6, IL-1β, and more without impeding the expression of the encoded antigens (234, 235). Although these responses can act to potentially enhance vaccine responses, excessive inflammation from unmodified mRNA can also promote RNA degradation (236). To overcome this, nucleoside-modified mRNAs, including pseudouridine incorporation, can help overcome such excessive responses and are commonly used in mRNA vaccine development (237). Further work is needed to determine the optimal balance of the intrinsic ability of vaccine mRNA to act as an adjuvant.

Similarly, DNA plasmids used in vaccine design have their own adjuvant-like capabilities through the activation of innate immune system DNA sensors. DNA plasmids used in these vaccines to encode an antigen of interest are derived from bacterial species. This bacterial DNA contains unmethylated CpG dinucleotide motifs bordered by two 3’ pyrimidines and 5’ purines, which can trigger the innate immune receptor TLR9 to activate the MyD88 signaling pathway resulting in pro-inflammatory cytokine release (238). As a result, when these plasmids are used for DNA vaccination, innate responses from APCs and other cells that take up the plasmid result in IL-6, IL-12, and IFNγ production. These responses were shown to be a significant factor in promoting DNA vaccine immunogenicity *in vivo*, indicating the advantageous self-adjuvating properties of DNA vaccines (239).

### Delivery system components

The second category of mRNA vaccine adjuvants includes the delivery system components. Because mRNA on its own is susceptible to extracellular degradation within the body and is unable to efficiently enter cells due to its negatively charged backbone, specific delivery systems are necessary. As the most used and optimal delivery platform for mRNA vaccines, lipid nanoparticles (LNPs) have been widely investigated for their immunostimulatory properties to enhance antigen-specific responses. Briefly, LNP-encapsulated mRNA is produced through the combination of cationic and structural lipids that complex with negatively charged mRNA molecules (240). Depending on the LNP, certain lipid components with adjuvating properties can be incorporated. The ionizable cationic lipid DLinDMA, for example, can be incorporated to induce potent germinal center Tfh and B cell responses to an mRNA-encoded antigen, leading to the production of neutralizing antibodies (169, 170, 241). As these responses are impaired in the elderly, targeted enhancement of GC Tfh and B cell responses offers an encouraging approach for enhancing aged vaccine responses. Additionally, a lipid-coated calcium phosphate nanoparticle can be applied to induce CD80/86 upregulation for enhanced DC maturation, further promoting improved antigen-specific responses following mRNA vaccination (172). Separately, the quaternary ammonium lipid DDA can be incorporated to enhance innate immunity and Th1 cell responses to vaccination when tested in a rabies mRNA vaccine model (173). At a higher level, the impact of the LNP component from SARS-CoV-2 mRNA vaccines was investigated in cells from young and aged individuals using an empty LNP (eLNP) (242). eLNP was found to induce maturation of monocyte derived DCs and upregulated CD40 and cytokine production (e.g. IFNγ) from DC and monocyte subsets in cells from both age groups, although to a lesser extent in cells from aged donors. Interestingly, eLNP was able to rescue phagocytic capacity in aged DCs. The aforementioned lipid components of LNPs for mRNA vaccine delivery represent only a fraction of the many components currently being investigated for adjuvant-like properties and offer promising directions for improving mRNA vaccine-elicited responses in elderly populations.

In the context of DNA vaccines, the diverse range of delivery systems includes many adjuvant-like properties. As previously discussed, external stimulation methods for DNA vaccine delivery largely stimulate immune responses through inflammatory induction to improve immunogenicity. For example, electroporation sends electrical pulses that induce danger signals from localized cell death and tissue damage, attracting APCs and lymphocytes to the site of vaccination for effective induction of the immune response, particularly in T cells, against the antigen of interest (186, 187). These local inflammatory responses recruit APCs and T cells through pro-inflammatory cytokine release, including TNFα and IL-1β (144). Electroporation not only improves cellular immune responses but can also lengthen the duration and array of responses to plasmid-encoded antigens (188). Studies have shown electroporation alone can increase immune responses to vaccination by 10- to 1000-fold (189, 190). Delivery devices can also stimulate the immunogenicity of DNA vaccines. The gene gun promotes a more specific Th2-biased response, but inferior cell-mediated immunity and limited capacity of DNA carried on the gold particles has resulted in its limited use (191, 192). Needle-free injections with next-generation jet injection devices have also shown to elicit dose-sparing vaccine responses, indicating improved immunogenicity; however, the specific causes of this have not been fully elucidated (243, 244).

Furthermore, like emerging formulations of mRNA vaccines, nanoparticles for DNA vaccine delivery have shown great potential for adjuvant capabilities outside of their function in DNA delivery. Chitosan, a cationic polymer that can form a protective nanoparticle around DNA vaccine plasmids, can enhance humoral and cellular responses to vaccination (193, 194) in addition to cytokine production (IFNγ), macrophage activation, and T cell responses (245). PLGA, another polymer used in DNA vaccine nanocarriers, has been found to improve MHC-I antigen presentation and T cell cytokine production (246). Additionally, studies of exosomes for DNA delivery have found the potential for deriving cell type-specific exosomes, such as DCs and other immune cells, to elicit intrinsic adjuvant effects following DNA vaccination (247).

### Exogenous adjuvants

Lastly, exogenous adjuvants for mRNA-based vaccines represent another method to boost vaccine responses. For example, arginine-rich protamine peptides can be applied to form a complex with mRNA to enhance TLR7/8 activation and promote B and T cell responses in a young and aged mouse model as well as in humans (108, 179). The use of the cholesterol-modified cationic peptide DP7 was found to enhance CD103+ DC maturation for heightened antigen presentation in mice (178). Exogenous adjuvants can also be delivered by encoding them in separate mRNA molecules administered during vaccination. In a proof-of-concept study, mice vaccinated against mRNA-encoded HPV E6 and E7 oncoproteins in combination with mRNA-encoded constitutively active STING showed reduced HPV+ TC-1 tumor growth and extended survival. This was linked to STING-mediated activation of type I interferon responses and enhanced antigen-specific T cell responses (180). Beyond these adjuvants, investigations into encoding additional proteins that are separate or linked to the encoded antigen show promising immunostimulatory properties. For example, the conjugation of human Fc to the receptor binding domain of SARS-CoV-2 encoded within a single mRNA molecule can promote recognition by Fc receptors on APCs to elicit enhanced vaccine responses (248). Interestingly, a more recent study that included mRNA-encoded IL-12p70 in a COVID-19 mRNA vaccine formulation found that the addition of the encoded cytokine significantly improved both humoral and cellular responses in aged mice, comparable to those seen in young (183). Such modifications offer promising new directions for enhancing the adjuvant-like properties of mRNA and their encoded antigens to improve protective responses to mRNA-based vaccines for vulnerable populations, such as the elderly.

Several adjuvants are available for use in both mRNA and DNA vaccine formulations. AS03 is a commercially available oil-in-water emulsion adjuvant that promotes both Th1 and Th2 responses for DNA and mRNA vaccinations (184, 249). Conversely, aluminum salts, or alum, can also be applied to these nucleic acid vaccine platforms for Th2-biased responses by enhancing antigen presentation through the absorption of antigens on their surface, activation of the NLRP3 inflammasome to generate IL-1β and IL-18, and activation of DCs through cytotoxic effects (226).

Both mRNA and DNA vaccines further enable adjuvants, such as cytokines, to be encoded along with the antigen of interest. Depending on the disease of interest, specific cytokines may be chosen to direct responses. For example, cell-mediated responses may be targeted by encoding IL-2, IL-12, IL-15, IL-18, or IFNγ (208, 209, 250). In the context of DNA vaccines, investigation into chemokines such as IL-8 (210), MIP-1α (251, 252), CCL28 (211), CCR10L (212), and others have shown to aid in DNA vaccine effectiveness *in vivo*. Other plasmid-encoded mediators like PD-1 have been used to promote CD8+ T cell responses to vaccination (220, 221). Beyond this, more recent investigations into plasmid-encoded adenosine deaminase-1 as a molecular adjuvant revealed improved antibody neutralization and durability, antigen-specific T cell cytokine production and polyfunctionality, and induction of Tfh-inducing cytokines, including IL-6 (222–225). While the list of molecular adjuvants for mRNA and DNA vaccines continues to expand, the ease of incorporating and investigating new adjuvants in nucleic acid vaccines highlights the potential for enhancing targeted immune responses to improve protection, particularly in immunocompromised individuals like the elderly. The selection of adjuvants enables the tailoring of immune responses to meet therapeutic requirements for the disease of interest.

Vaccine adjuvants are particularly critical to induce protective immune responses in elderly populations, and although several have been investigated in the context of aging including AS01 and MF59, many of these adjuvants have not been applied to nucleic acid vaccine platforms. With limited work on vaccine adjuvants in this context, it is evident that extensive work remains to be done on mRNA and DNA vaccine adjuvant efficacy and development in the aged.

## Future directions of nucleic acid-based vaccines

Further investigations into pre-clinical mRNA and DNA vaccines currently lacking aged models are still necessary to guide vaccine development for the elderly. In the rapidly growing field of mRNA vaccines, there remain several infectious agents not previously discussed that are being targeted in early investigations. The University of Pennsylvania, widely known for its role in successfully developing the first clinically used mRNA vaccine against COVID-19, is at the forefront for many new mRNA vaccine formulations that are undergoing investigation. Although they remain to be tested in aging models, infectious diseases that disproportionately impact elderly individuals including Avian Bird Flu (253), *Clostridioides difficile* (254), Norovirus, and Tuberculosis are being targeted with novel mRNA formulations (255). Similarly, DNA vaccines targeting *C. difficile* (256, 257), *Streptococcus pneumoniae* (258), and RSV (259) exist but have not been tested in aging. Incorporation of aged models, particularly for pathogens that significantly impact elderly populations, could provide insight into the potential for nucleic acid vaccines to induce protective immune responses against targets where previously tested vaccines have fallen short.

Newer developments in self-amplifying mRNA (saRNA) are also paving the way for the future of nucleic acid vaccine design. Despite first being described in 2012 (260), saRNA has gained more attention since the implementation of the COVID-19 mRNA vaccine. This emerging mRNA technology, unlike conventional mRNA vaccines, incorporates encoded viral replicase genes in addition to the antigen of interest to promote self-amplification of the mRNA, thereby rapidly increasing production of antigen. This self-amplifying property overcomes obstacles observed in conventional mRNA vaccines including balancing high doses with adverse side effects, stability, and the need for multiple boosting vaccinations. Based on their potent induction of immune responses at lower vaccination doses (261), saRNA vaccine technology is primed for investigation aging models. In fact, recent approval of the first saRNA vaccine in Japan, which targets COVID-19, highlights the potential of this new mRNA vaccine technology and the need for evaluation of immune responses elicited in elderly populations (262).

Finally, this review highlights the shortcomings of clinical trials examining the efficacy of nucleic acid vaccines and adjuvants in aged individuals. A greater focus on age-related deficits following nucleic acid vaccination are critically needed to identify and correct vaccine design for improved responses in this susceptible population.
